# Interleukin-10 −592C/A, −819C/T and −1082A/G Polymorphisms with Risk of Type 2 Diabetes Mellitus: A HuGE Review and Meta-analysis

**DOI:** 10.1371/journal.pone.0066568

**Published:** 2013-06-21

**Authors:** Yanyin Hua, Jie Shen, Yingxiang Song, Yubo Xing, Xiao Ye

**Affiliations:** Department of Endocrinology, Zhejiang Provincial People’s Hospital, Hangzhou, China; University of KwaZulu-Natal, South Africa

## Abstract

**Background:**

Several studies have been conducted in recent years to evaluate the risk of type 2 diabetes mellitus (T2DM) and polymorphisms of interleukin (IL)-10. However, the results remain conflicting rather than conclusive. This meta-analysis aimed to summarize the current evidence from case-control studies that evaluated this association.

**Methods:**

We carried out a search in Medline, EMBASE, and the Chinese National Knowledge Infrastructure (CNKI) database for relevant studies. Data were extracted using a standardized form and pooled odds ratios (ORs) with 95% confidence intervals (CIs) were calculated to assess the strength of the association.

**Results:**

10 studies were included in our meta-analysis and systemic review. Our meta-analysis indicated that IL-10 −1082A/G polymorphism was associated with the risk of T2DM (GA *vs.* AA: OR = 1.21, 95% CI = 1.03–1.14; GA/GG *vs.* AA: OR = 1.22, 95% CI = 1.05–1.41), whereas there was no association between IL-10 −592C/A (CC/CA *vs.* AA: OR = 1.07, 95% CI = 0.59–1.93) or -819C/T (CC/CT *vs.* TT: OR = 0.93, 95% CI = 0.49–1.75) polymorphism and T2DM risk was found in our study.

**Conclusions:**

This meta-analysis provides strong evidence that IL-10 −1082A/G polymorphism associated with risk of T2DM. However, no association of the IL-10 −592C/A or −819C/T polymorphism with T2DM risk was found. Additional well-designed large studies were required for the validation of our results.

## Introduction

Type 2 diabetes mellitus (T2DM) is one of the main chronic diseases and its complications have become a major cause of morbidity, mortality, and disability in the World. It has been estimated that the number of people with type 2 diabetes will double to at least 350 million worldwide by 2030 unless appropriate action is taken [1]. However, up to date, the mechanism of the disease is still not fully understood. In recent years, published data showed that genetic polymorphisms might explain individual differences in T2DM risk [2], [3]. Several candidate genes are implicated in the pathogenesis of T2DM, one of which is interleukin (IL)-10.

Exel and colleagues discovered that low IL-10 production capacity is associated with the metabolic syndrome and T2DM [4]. The capacity for IL-10 production in individuals has been shown to be correlated with genetic composition of the IL-10 locus [5]. Thus, examination of genetic polymorphisms of IL-10 may explain individual differences in T2DM risk. Several molecular epidemiological studies were conducted in recent years to evaluate the risk of T2DM associated with the polymorphisms of IL-10 [5]–[14]. However, the results remain conflicting rather than conclusive. Considering the relatively small sample size in each study, it is possible to perform a quantitative synthesis of the evidence with rigorous methods. To investigate a possible association between IL-10 −592C/A, −819C/T and −1082A/G polymorphisms and T2DM, we performed a meta-analysis from all of the available relevant studies.

## Materials and Methods

### Identification and Eligibility of Relevant Studies

We carried out a search in Medline, EMBASE, and Chinese National Knowledge Infrastructure (CNKI), covering all papers published up to Aug 2012, using the search terms: (“Interleukin-10” OR “IL-10”) AND (“gene” OR “polymorphism” OR “genetic variant”) AND (“type 2 diabetes mellitus” OR “T2DM” OR “diabetes mellitus” OR “type 2 diabetes”). All eligible studies were retrieved, and their bibliographies were checked for other relevant publications. Only published studies with full-text articles were included.

### Inclusion and Exclusion Criteria

The inclusion criteria were as follows: (a) published in English or in Chinese; (b) used a case-control design; (c) supplied the available genotype frequencies in cases and controls; and (d) sufficient published data for estimating an odds ratio (OR) with 95% confidence interval (CI). Major reasons for exclusion of studies were (a) no control group; (b) duplicate of previous publication.

### Data Extraction

Two investigators reviewed the articles independently to exclude irrelevant and overlapping studies. The results were compared, and disagreements were resolved by discussion and consensus. From each study, the following information was extracted: first author’s surname, year of publication, ethnic descent of the study population (European, Asian and African), definition of case, age, characteristics of controls, numbers of eligible cases and controls, and genotype distributions in cases and controls.

### Statistical Analysis

The Hardy-Weinberg equilibrium (HWE) was utilized to compare the observed genotype frequencies with expected genotype frequencies in controls for all studies. OR and 95% CI were used to assess the strength of association between IL-10 polymorphisms and the risk of T2DM under homozygote comparison, heterozygote comparison, dominant and recessive genetic model comparison. The significance of the combined OR was determined by the Z-test, in which *P*<0.05 was considered significant. Stratified analyses were performed by ethnicity, age and sources of control. The χ2-based Q statistical test was used for the assessment of the between-study heterogeneity, which was considered significant for *P*<0.1 [15]. In analyses, if the heterogeneity was low then we used a fixed-effect model, or else applied the random-effect model. Sensitivity analyses were also performed to assess the stability of the results [16]. Funnel plots and Egger’s linear regression test were used to provide diagnosis of the potential publication bias [17]. All analyses were performed with Stata (Version10.0, Stata Corporation) and Review Manager (version 5.0.16, The Cochrane Collaboration) using two sided *P* values.

## Results

### Characteristics of Studies

**Figure 1:**
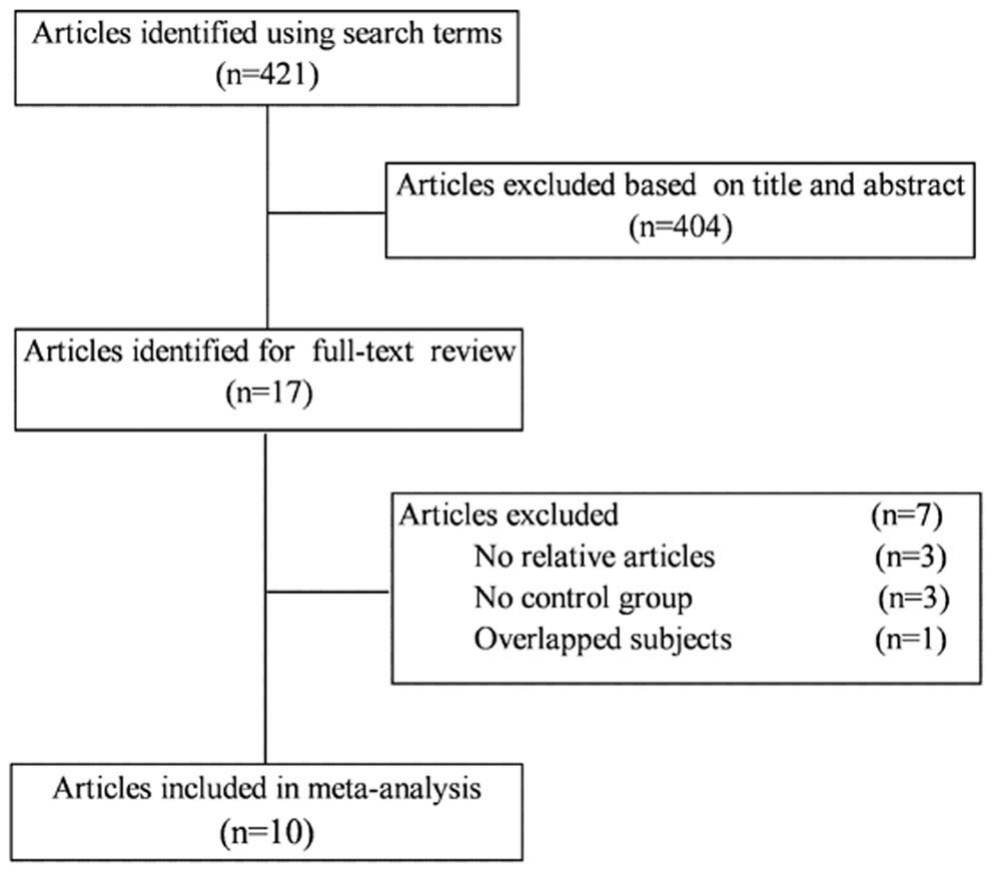
Flow diagram of the literature search and trial selection process.

The search initially identified 421 potentially eligible articles. Of these, the first screening excluded 404 citations based on abstracts or titles, leaving 17 articles for full-text review. The excluded 7 articles had no relative outcomes, no control group, and duplicate of previous publication. We finally included 10 studies in our systematic review and meta-analysis [5]–[14]. The detailed steps of our literature search are shown in Figure 1.

The characteristics of 10 included studies are summarized in Table 1. There are eight case-control studies concerning −592C/A polymorphism [5]–[12], four case-control studies concerning −819C/T polymorphism [5]–[8], and six case-control studies concerning −1082A/G polymorphism [6]–[9], [13], [14]. Controls were selected from healthy population in all the studies and most studies used frequency-matched controls to the cases by age, sex, residence, or ethnicity. The genotype distributions among the controls of all studies were in agreement with HWE except for three studies for the −592C/A [8], [10], [12], one study for the −819C/T [8], and two for the −1082A/G [8], [13].

**Table 1:** Study characteristics of inclulded studies in this meta-analysis.

Author	Year	Country	Ethnicity	Source of controls	SNPs studied	Sample size	HWE
Chang [5]	2005	China	Asian	Healthy Control	–592C/A, –819C/T	370/175	0.86, 0.87
Ezzidi [6]	2009	Tunisia	African	Healthy Control	–592C/A, –819C/T, –1082G/A	917/748	0.41, 0.41, 0.14
Tsiavou [7]	2004	Greece	European	Healthy Control	–592C/A, –819C/T, –1082G/A	31/39	0.38, 0.38, 0.82
Kung [8]	2010	China	Asian	Healthy Control	–592C/A, –819C/T, –1082G/A	47/25	<0.01, <0.01, <0.01
Scarpelli [9]	2006	Italy	European	Healthy Control	–592C/A, –1082G/A	551/1131	0.21, 0.68
Saxena [10]	2012	India	Asian	Healthy Control	–592C/A	406/168	0.01
Arababadi [11]	2012	Iran	Asian	Healthy Control	–592C/A	200/100	0.32
Wang [12]	2010	China	Asian	Healthy Control	–592C/A	224/275	0.04
Kolla [13]	2009	India	Asian	Healthy Control	–1082G/A	198/202	<0.01
Erdogan [14]	2012	Turkey	Asian	Healthy Control	–1082G/A	91/112	0.68

### Quantitative Synthesis

#### IL-10 -592C/A

**Figure 2:**
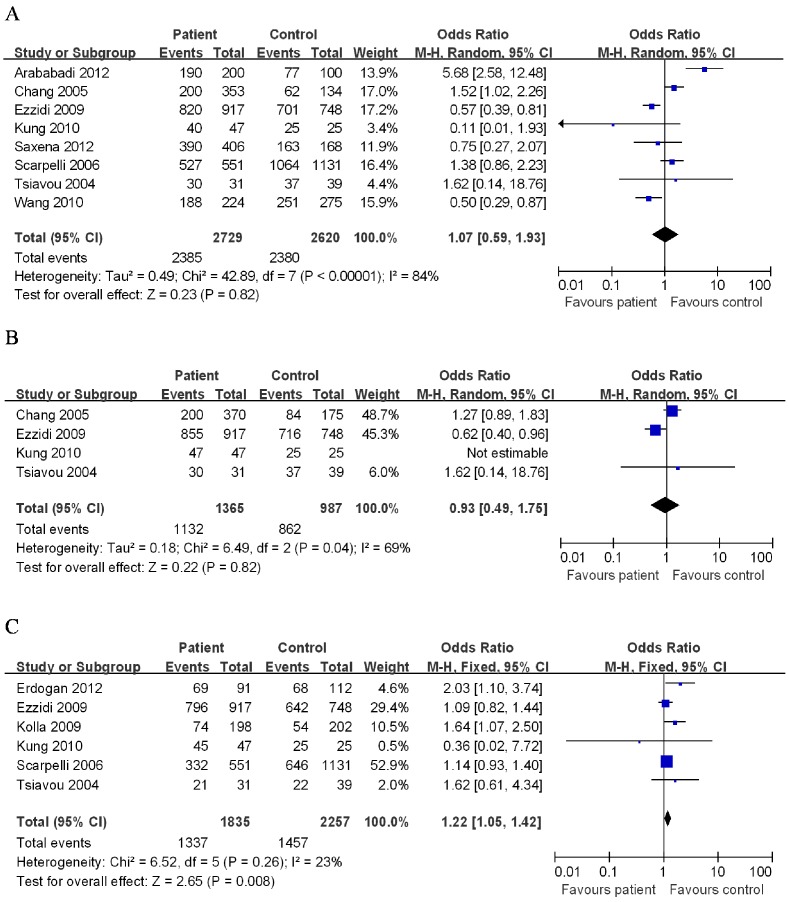
Meta-analysis with a random-effect model for the ORs of type 2 diabetes mellitus risk associated with interleukin-10 polymorphisms in dominant genetic model comparison. (A: −592C/A; B: −819C/T; C: −1082A/G.).

The evaluation of association between IL-10 polymorphisms and T2DM risk was presented in Table 2. Overall, no significant association was found between IL-10 −592C/A polymorphism and risk of T2DM (CC/CA *vs.* AA: OR = 1.07, 95% CI = 0.59–1.93; Figure 2A). We further performed a subgroup analysis by ethnicity and obtained statistically similar results in European descendents and Asian descendents. However, a statistically significant association of IL-10 −592C/A polymorphism and T2DM risk were found in African descendents (CC/CA *vs.* AA: OR = 0.58, 95% CI = 0.41–0.82),

**Table 2:** Total and stratified analyses of the interleukin-10 polymorphisms on type 2 diabetes mellitus risk.

		Cases/	Homozygote		Heterozygote		Dominant Model		Recessive Model	
Variables	No.^a^	Controls	OR(95%CI)	*P* ^b^	OR(95%CI)	*P* ^b^	OR(95%CI)	*P* ^b^	OR(95%CI)	*P* ^b^
**–592C/A**	8	2729/2620	1.17[0.54,2.55]	0.00^c^	1.05[0.66,1.68]	0.00^c^	1.07[0.59,1.93]	0.00^c^	1.13[0.79,1.61]	0.00^c^
**European**	2	582/1170	1.36[0.86,2.13]	0.84	1.38[0.87,2.18]	0.99	1.37[0.88,2.12]	0.90	1.02[0.84,1.25]	0.64
**Asian**	5	1203/702	1.27[0.32,5.05]	0.00^c^	1.08[0.52,2.23]	0.00^c^	1.11[0.44,2.80]	0.00^c^	1.39[0.65,2.96]	0.00^c^
**African**	1	917/748	0.53[0.37,0.75]	NA	0.65[0.45,0.94]	NA	0.58[0.41,0.82]	NA	0.74[0.61,0.90]	NA
**–819C/T**	4	1365/987	0.99[0.37,2.69]	0.01^c^	1.02[0.77,1.36]	0.41	0.93[0.49,1.75]	0.04^c^	0.93[0.49,1.78]	0.03^c^
**European**	1	31/39	1.72[0.17,17.75]	NA	1.41[0.13,15.13]	NA	1.58[0.16,15.94]	NA	1.27[0.50,3.25]	NA
**Asian**	2	417/200	1.63[0.87,3.03]	NA	1.20[0.82,1.75]	NA	1.27[0.89,1.83]	NA	1.40[0.77,2.55]	0.12
**African**	1	917/748	0.54[0.35,0.82]	NA	0.81[0.52,1.27]	NA	0.63[0.41,0.95]	NA	0.64[0.52,0.77]	NA
**–1082G/A**	6	1835/2257	1.37[0.84,2.25]	0.01^c^	1.21[1.03,1.14]	0.19	1.22[1.05,1.41]	0.26	1.25[0.76,2.03]	0.00^c^
**European**	2	582/1170	1.22[0.88,1.69]	0.24	1.14[0.92,1.41]	0.81	1.15[0.94,1.41]	0.49	1.14[0.84,1.55]	0.23
**Asian**	3	336/339	0.56[0.01,29.63]	0.00^c^	1.60[0.81,3.17]	0.12	1.69[1.21,2.38]	0.33	0.38[0.00,47.72]	0.00^c^
**African**	1	917/748	1.03[0.76,1.39]	NA	1.15[0.85,1.54]	NA	1.09[0.82,1.44]	NA	0.92[0.76,1.13]	NA

a numberof studies; b *P* value of Q-test for heterogeneity test; c Random-effects model was used when *P* value for heterogeneity test <0.10;

otherwise, fixed-effects model was used; 0.00 means value <0.01; NA not applicable.

#### IL-10−819C/T

There was no statistically significant differences between IL-10 −819C/T polymorphism and T2DM risk (CC/CT *vs.* TT: OR = 0.93, 95% CI = 0.49–1.75; Figure 2B). In the stratified analyses for the −819C/T polymorphism, there was a significantly increased risk was observed among African descendents (CC/CT *vs.* TT: OR = 0.63, 95% CI = 0.41–0.95), while these association were not found in European descendents or Asian descendents in the four tested models.

#### IL-10−1082A/G

Significant association between IL-10 −1082A/G polymorphism and risk of T2DM was observed under heterozygote comparison (GA *vs.* AA: OR = 1.21, 95% CI = 1.03–1.14) and dominant genetic model (GA/GG *vs.* AA: OR = 1.22, 95% CI = 1.05–1.41; Figure 2C). In the stratified analysis by ethnicity, IL-10 −1082A/G polymorphism was associated with a significantly increased risk of T2DM in Asian descendents under dominant genetic model (GA/GG *vs.* AA: OR = 1.69, 95% CI = 1.21–2.38). However, no significant association was found in European descendents or African descendents in all tested models.

### Test of Heterogeneity

There was significant heterogeneity in our meta-analysis. We then assessed the source of heterogeneity for homozygote comparison by ethnicity and sample size. As a result, for −592C/A and −819C/T, ethnicity (*x^2^* = 12.32, *P* = 0.002; *x^2^* = 8.46, *P* = 0.01; respectively), but not sample size (*x^2^* = 1.10, *P* = 0.29; *x^2^* = 0.73, *P* = 0.39; respectively), was found to contribute to substantial heterogeneity. However, neither ethnicity (*x^2^* = 1.77, *P* = 0.41) nor sample size(*x^2^* = 2.34, *P* = 0.13) was found to contribute to substantial heterogeneity for −1082A/G.

### Sensitivity Analysis

Although the distribution of genotypes in the controls in some studies did not follow HWE, the corresponding pooled OR and between-study heterogeneity were not significant altered without these studies (CC *vs*. AA: OR = 1.87, 95% CI = 0.66–5.30, *P*_heterogeneity_ <0.01 for −592C/A; CC *vs*. CT/TT: OR = 1.00, 95% CI = 0.51–1.96, *P*_heterogeneity_ = 0.02 for −819C/T). However, sensitivity analysis indicated the studies by Kung et al. and Kolla et al. were the main origin of heterogeneity for −1082A/G. The heterogeneity significantly decreased when excluding the two studies (*P*_heterogeneity_ = 0.13), while the value of pooled OR was not significantly altered without the two studies (GG *vs*. AA OR = 1.11, 95% CI = 0.74–1.65).

### Publication Bias

**Figure 3:**
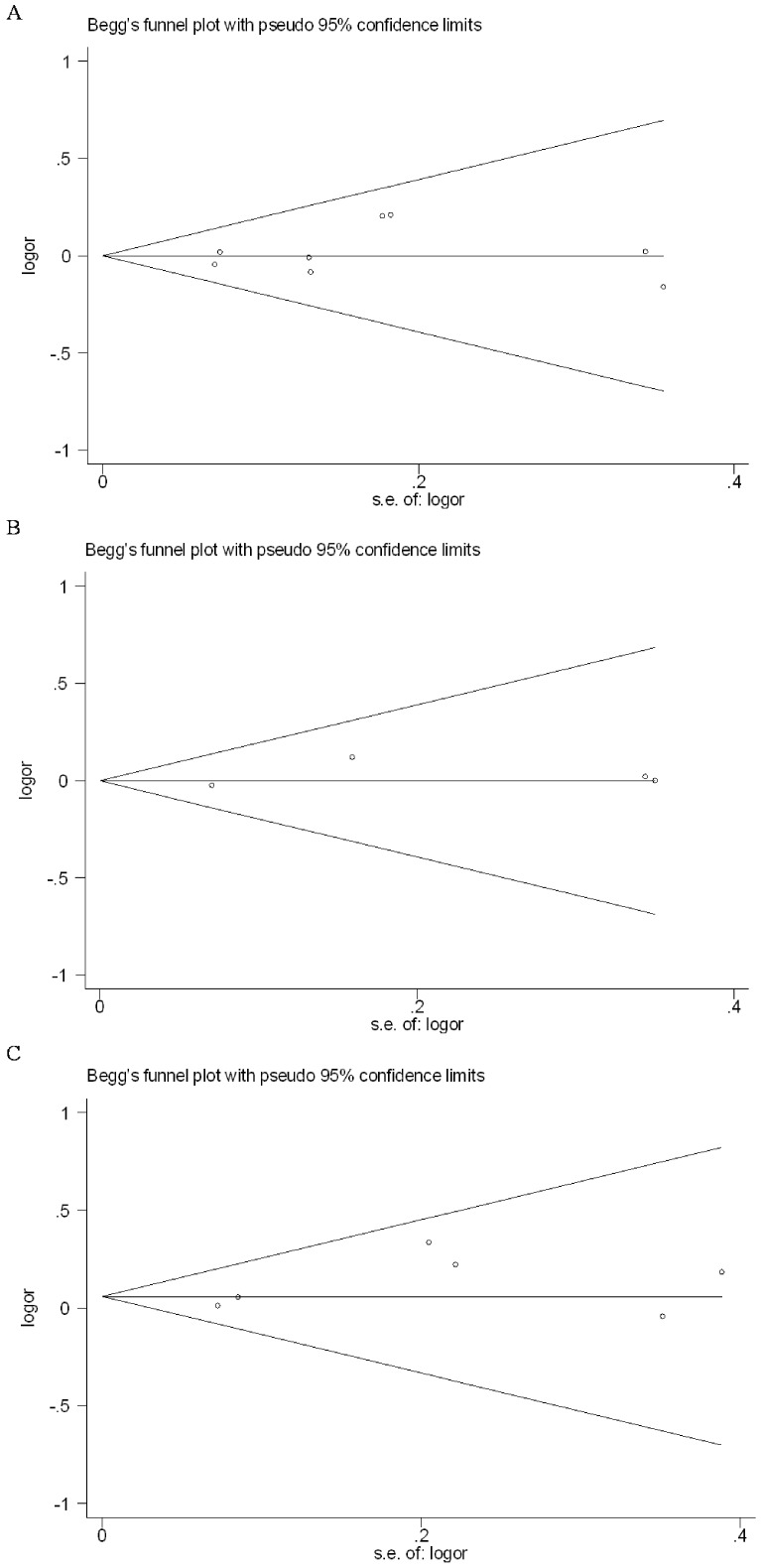
Funnel plot for publication bias of the meta-analysis of type 2 diabetes mellitus risk and interleukin-10 polymorphisms in dominant genetic model comparison. (A: −592C/A; B: −819C/T; C: −1082A/G.).

We used Funnel plot and Egger’s regression asymmetry test to access the publication bias of literatures. The data suggested that there was no evidence of publication bias in dominant genetic model comparison (t = 0.74, *P = *0.486 for −592C/A; t = 0.80, *P = *0.507 for −819C/T; t = 1.49, *P = *0.209 for −1082A/G; Figure 3).

## Discussion

In the present study, a meta-analysis was performed to examine the association between three IL-10 polymorphisms and T2DM risk, by critically reviewing 8 studies on IL-10 −592C/A polymorphism (2,729 patients and 2,620 controls), 4 studies on IL-10 −819C/T polymorphism (1,365 patients and 987 controls), and 6 studies on IL-10 −1082A/G polymorphism (1,835 patients and 2,257 controls). To the best of our knowledge, this is the first comprehensive meta-analysis to date investigating the association between IL-10 −819C/T and −1082A/G polymorphisms and T2DM risk. In addition, more studies were included in our study than a recently published meta-analysis concerning −592C/polymorphisms and T2DM risk [18].

The findings from our study indicated that IL-10 −1082A/G polymorphism associated with risk of T2DM. However, no association of the IL-10 −592C/A or −819C/T polymorphism with T2DM risk was found. There was evidence of heterogeneity between studies in our analyses. We then assessed the source of heterogeneity for homozygote comparison by ethnicity and sample size. As a result, ethnicity was found to contribute to heterogeneity for IL-10 −592C/A and −819C/T polymorphisms. However, the reasons for heterogeneity in the analysis of −1082A/G were unclear. It may be due to other factors, including the selection of methods, definition of cases, and sample sizes [19]. We further performed a sensitivity analysis and found the studies by Kung et al. [8] and Kolla et al. [13] were the main origin of heterogeneity for −1082A/G.

Allele frequency might reflect the natural selection pressures or a balance by other related functional genetic variants and environmental exposures [20], [21]. Thus, a subgroup analysis by ethnicity was performed in our meta-analyses. In stratified analyses, we observed a significant association between IL-10 −592C/A and −819C/T polymorphisms and T2DM risk in Africans. At the same time, it is worth emphasizing that IL-10 −1082A/G polymorphism contributed to an increase in T2DM susceptibility in Asians, but not for Europeans and Africans. Different genetic background and environmental exposures might contribute to this ethnic difference. However, there are only one study included in the analysis of African descendents with limited sample sizes, the result should be interpreted with caution, therefore, more studies based on larger population should be conducted to further examine those associations.

There are some limitations of this meta-analysis should be acknowledged. Firstly, detailed information, such as environmental exposures and gene-gene interactions, were unavailable in most studies, which limited our further assessment of those confounding factors at the patient level, and were incorporated into the analysis. Secondly, some studies with small sample size appear to overestimate the true association due to lack of sufficient power to detect such an association. Thirdly, only English and Chinese language studies were included in this meta-analysis might have led to bias.

Even though, our meta-analysis has some advantages. Firstly, the search and selection studies were conducted very strictly, which significantly increased statistic power of this meta-analysis. Secondly, all the studies included in this meta-analysis were case-control researches and contained available genotype frequency, which met our including criterion well. Third, controls in all the included studies were healthy population, which avoided occurring population stratification bias.

In conclusion, our meta-analysis suggested that IL-10 −592C/A or −819C/T polymorphism had no association with T2DM risk in all examined patients, whereas there was an association between IL-10 −1082A/G polymorphism and risk of T2DM. However, additional large studies are warranted to validate our findings. Future studies should include multi-ethnic groups and use standardized unbiased genotyping methods, different grades of asthma patients, and well-matched controls.

## References

[1] Screening for Type 2 Diabetes: Report of a World Health Organization and International Diabetes Federation meeting. Available: http://www.who.int/diabetes/publications/en/screening_mnc03.pdf. Accessed 2002 May 9–11.

[2] Biswas D, Vettriselvi V, Choudhury J, Jothimalar R (2011) Adiponectin gene polymorphism and its association with type 2 diabetes mellitus. Indian J Clin Biochem 26: 172–177. 22468045 10.1007/s12291-011-0123-5 PMC3107406

[3] Perez-Luque E, Malacara JM, Garay-Sevilla ME, Fajardo ME (2012) Association of the TNF-α -308G/A polymorphism with family history of type 2 diabetes mellitus in a Mexican population. Clin Biochem 45: 12–15. 22015686 10.1016/j.clinbiochem.2011.09.018

[4] van Exel E, Gussekloo J, de Craen AJ, Frölich M, Bootsma-Van Der Wiel A, et al (2002) Low production capacity of interleukin-10 associates with the metabolic syndrome and type 2 diabetes: the Leiden 85-Plus Study. Diabetes 51: 1088–1092. 11916930 10.2337/diabetes.51.4.1088

[5] Chang YH, Huang CN, Wu CY, Shiau MY (2005) Association of interleukin-10 A-592C and T-819C polymorphisms with type 2 diabetes mellitus. Hum Immunol 66: 1258–1263. 16690414 10.1016/j.humimm.2005.05.001

[6] Ezzidi I, Mtiraoui N, Kacem M, Mallat SG, Mohamed MB, et al (2009) Interleukin-10–592C/A, −819C/T and −1082A/G promoter variants affect the susceptibility to nephropathy in Tunisian type 2 diabetes (T2DM) patients. Clin Endocrinol (Oxf) 70: 401–407. 18616700 10.1111/j.1365-2265.2008.03337.x

[7] Tsiavou A, Hatziagelaki E, Chaidaroglou A, Manginas A, Koniavitou K, et al (2004) TNF-alpha, TGF-beta1, IL-10, IL-6, gene polymorphisms in latent autoimmune diabetes of adults (LADA) and type 2 diabetes mellitus. J Clin Immunol 24: 591–599. 15622443 10.1007/s10875-004-6239-0

[8] Kung WJ, Lin CC, Liu SH, Chaung HC (2010) Association of interleukin-10 polymorphisms with cytokines in type 2 diabetic nephropathy. Diabetes Technol Ther 12: 809–813. 20809684 10.1089/dia.2010.0085

[9] Scarpelli D, Cardellini M, Andreozzi F, Laratta E, Hribal ML, et al (2006) Variants of the interleukin-10 promoter gene are associated with obesity and insulin resistance but not type 2 diabetes in caucasian italian subjects. Diabetes 55: 1529–1533. 16644716 10.2337/db06-0047

[10] Saxena M, Agrawal CG, Bid HK, Banerjee M (2012) An Interleukin-10 Gene Promoter Polymorphism (−592A/C) Associated with Type 2 Diabetes: A Nor.th Indian Study. Biochem Genet *In Press*. 10.1007/s10528-012-9499-z 22298356

[11] Arababadi MK, Reza Mirzaei M, Hassanshahi G, Ahmadabadi BN, Ahmadabadi BN, et al (2012) Interleukin (IL)-10 gene polymorphisms are associated with type 2 diabetes with and without nephropathy: a study of patients from the southeast region of Iran. Inflammation 35: 797–802. 21909800 10.1007/s10753-011-9376-7

[12] Wang JD, Fang H, Yan QZ, Zhou DH, Yao HJ (2013) Relationship of Serum laterleuldn-10 Level and Its Gem Promoter 592 Polymorphism to Type 2 Diabetes Mellitus. Chinese General Practice 13: 1185–1188.

[13] Kolla VK, Madhavi G, Pulla Reddy B, Srikanth Babu BM, Yashovanthi J, et al (2009) Association of tumor necrosis factor alpha, interferon gamma and interleukin 10 gene polymorphisms with peripheral neuropathy in South Indian patients with type 2 diabetes. Cytokine 47: 173–177. 19608431 10.1016/j.cyto.2009.06.007

[14] Erdogan M, Cetinkalp S, Ozgen AG, Saygili F, Berdeli A, et al (2012) Interleukin-10 (−1082G/A) gene polymorphism in patients with type 2 diabetes with and without nephropathy. Genet Test Mol Biomarkers 16: 91–94. 21861711 10.1089/gtmb.2011.0075

[15] Cao C, Wang J, Bunjhoo H, Xu Y, Fang H (2012) Risk profile of bevacizumab in patients with non-small cell lung cancer: a meta-analysis of randomized controlled trials. Acta Oncol 51: 151–156. 22085338 10.3109/0284186X.2011.631579

[16] Landbo C, Prescott E, Lange P, Vestbo J, Almdal TP (1999) Prognostic value of nutritional status in chronic obstructive pulmonary disease. Am J Respir Crit Care Med 160: 1856–1861. 10588597 10.1164/ajrccm.160.6.9902115

[17] Egger M, Davey Smith G, Schneider M, Minder C (1997) Bias in meta analysis detected by a simple, graphical test. BMJ 315: 629–634. 9310563 10.1136/bmj.315.7109.629 PMC2127453

[18] Yin YW, Sun QQ, Zhang BB, Hu AM, Liu HL, et al (2012) Association between interleukin-10 gene −592 C/A polymorphism and the risk of type 2 diabetes mellitus: A meta-analysis of 5320 subjects. Hum Immunol 73: 960–965. 22732092 10.1016/j.humimm.2012.06.006

[19] Cao C, Ying T, Fang JJ, Sun SF, Lv D, et al (2011) Polymorphism of vascular endothelial growth factor −2578C/A with cancer risk: evidence from 11263 subjects. Med Oncol 28: 1169–1175. 20635170 10.1007/s12032-010-9613-1

[20] Zhang YM, Cao C, Liang K (2010) Genetic polymorphism of epidermal growth factor 61A>G and cancer risk: a meta-analysis. Cancer Epidemiol 34: 150–156. 20207214 10.1016/j.canep.2010.02.004

[21] Cao C, Fang JJ, Ying T, Sun SF, Lv D, et al (2010) Vascular endothelial growth factor +936C/T and +405G/C polymorphisms and cancer risk: a meta-analysis. Arch Med Res 41: 548–557. 21167395 10.1016/j.arcmed.2010.09.006

